# Antioxidant Nanozymes: Mechanisms, Activity Manipulation, and Applications

**DOI:** 10.3390/mi14051017

**Published:** 2023-05-09

**Authors:** Nguyen Thi My Thao, Hoang Dang Khoa Do, Nguyen Nhat Nam, Nguyen Khoi Song Tran, Thach Thi Dan, Kieu The Loan Trinh

**Affiliations:** 1 School of Medicine and Pharmacy, Tra Vinh University, Tra Vinh City 87000, Vietnam; 2 NTT Hi-Tech Institute, Nguyen Tat Thanh University, Ward 13, District 04, Ho Chi Minh City 70000, Vietnam; 3 Biotechnology Center, School of Agriculture and Aquaculture, Tra Vinh University, Tra Vinh City 87000, Vietnam; 4 College of Korean Medicine, Gachon University, 1342 Seongnam-daero, Sujeong-gu, Seongnam-si 13120, Republic of Korea; tsnkhoi@gmail.com; 5 Tra Vinh University, Tra Vinh City 87000, Vietnam; 6 Department of BioNano Technology, Gachon University, 1342 Seongnam-daero, Sujeong-gu, Seongnam-si 13120, Republic of Korea

**Keywords:** antioxidant nanozyme, catalase, superoxide dismutase, glutathione peroxidase, enzymatic manipulation

## Abstract

Antioxidant enzymes such as catalase, superoxide dismutase, and glutathione peroxidase play important roles in the inhibition of oxidative-damage-related pathological diseases. However, natural antioxidant enzymes face some limitations, including low stability, high cost, and less flexibility. Recently, antioxidant nanozymes have emerged as promising materials to replace natural antioxidant enzymes for their stability, cost savings, and flexible design. The present review firstly discusses the mechanisms of antioxidant nanozymes, focusing on catalase-, superoxide dismutase-, and glutathione peroxidase-like activities. Then, we summarize the main strategies for the manipulation of antioxidant nanozymes based on their size, morphology, composition, surface modification, and modification with a metal-organic framework. Furthermore, the applications of antioxidant nanozymes in medicine and healthcare are also discussed as potential biological applications. In brief, this review provides useful information for the further development of antioxidant nanozymes, offering opportunities to improve current limitations and expand the application of antioxidant nanozymes.

## 1. Introduction

During the metabolism processes and normal physiological activities of most aerobic organisms, oxygen undergoes a series of chemical reactions resulting in the formation of toxic by-products, such as superoxide anion radicals (O_2_^•−^), hydroxyl radicals, and hydrogen peroxide (H_2_O_2_). These by-products are called reactive oxygen species (ROS) and are known as one of the main causes of oxidative damage to some important biological molecules (e.g., DNAs, RNAs, lipids, and proteins) [1,2]. Although ROS are required for numerous essential functions, such as signaling cascade and redox-governing activities, an excess of ROS can cause severe diseases, such as neurodegenerative diseases, digestive diseases, respiratory diseases, and cancer [3,4]. To balance the level of ROS, organisms build an antioxidant system comprised of antioxidant enzymes that can neutralize or degrade the excessive ROS. The natural antioxidant enzymes mainly include catalase (CAT), superoxide dismutase (SOD), and glutathione peroxidase (GPx). While antioxidant enzymes have been widely used to inhibit oxidative damage-related diseases, they have certain limitations, such as high cost, low stability, difficult storage, and poor reusability [5,6,7].

**Figure 1:**
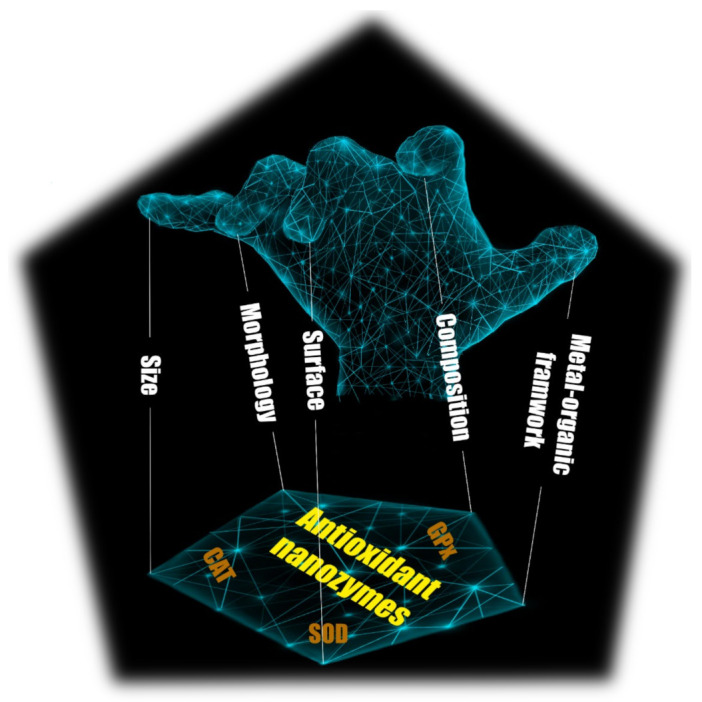
Manipulation of antioxidant nanozymes exhibiting CAT-, SOD-, and GPx-like activity.

Taking advantage of nanotechnology, nanozymes have emerged as a neoteric approach to improve the limitations of natural antioxidant enzymes. Nanozymes are known as materials with sizes of 100 nm or smaller that display enzyme-like activities. Similar to natural antioxidant enzymes, antioxidant nanozymes catalyze the degradation or decomposition of oxidative agents. In addition, antioxidant nanozymes tend to become popular and promising artificial enzymes owing to their advantages. First, nanozymes can be easily manufactured and do not require expensive storage compared to natural enzymes [8]. Second, the activities of nanozymes can be rationally designed [9]. Third, nanozymes are supposed to be more stable and durable [10]. The mechanisms of antioxidant nanozymes mainly rely on intrinsic CAT, SOD, and GPx mimics to decrease the level of ROS [11,12,13]. The antioxidant activities of nanozymes are highly affected by various factors, including the size and morphology of materials, surface modification, composition, etc. Although dozens of excellent reviewers have discussed the mechanisms and regulation of nanozymes, an overview of antioxidant nanozyme profiles is still lacking. Therefore, in this review, we first discuss the three key mechanisms of antioxidant nanozymes, which are CAT, SOD, and GPx activities (Figure 1). Afterward, we summarize the factors for the manipulation of antioxidant nanozymes. Finally, we review the application of antioxidant nanozymes in several fields, such as medicine, healthcare, diagnostics, and analytics.

## 2. Mechanisms of Nanozymes for Antioxidant Effects

### 2.1. Catalase-Like Activity

Catalases (CAT) are natural enzymes containing iron porphyrin at their active sites and are known as antioxidant enzymes because they can catalyze the degradation of hydrogen peroxide to form molecular oxygen and water [14,15,16]. Although hydrogen peroxides play an important role in biological systems, an excess of hydrogen peroxides in the cytoplasm can undergo the Fenton reaction in the presence of transition metal ions and generate hydroxyl radicals, which are strong oxidants [17,18]. Thanks to the feature of CATs that guards living systems against potential oxidative damage, two hydrogen peroxide molecules are degraded to generate two water molecules and one oxygen molecule in a two-step reaction. First, the iron porphyrin group (Fe^3+^) of CAT reacts with one hydrogen peroxide to form an oxoferryl porphyrin cation radical (Fe^4+^). The radical then degrades a second hydrogen peroxide to generate water and oxygen molecules, while Fe^4+^ is reduced back to the Fe^3+^ state [19,20].

(1) $$2H_{2}O_{2}\overset{Catalase}{\to }{\;O}_{2}+2H_{2}O$$

**Figure 2:**
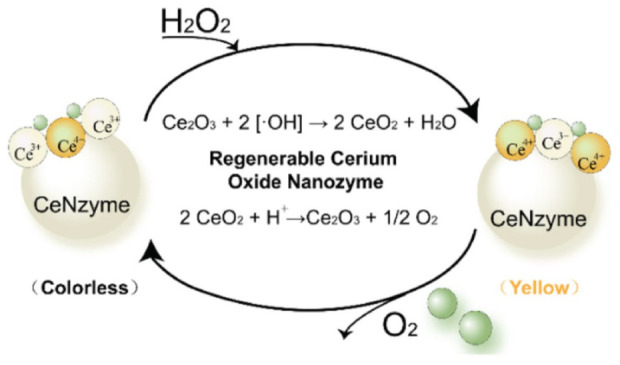
Cerium-based nanozyme exhibited CAT-like activity through cycling between Ce^4+^ and Ce^3+^ oxidation states. Reprinted with permission from [28]. Copyright (2020) ACS publications.

Inspired by the nature of CATs, metal/metal-oxide-based nanomaterials, such as cerium oxide, cobalt oxide, iron oxide, and gold nanoparticles, have been investigated to mimic CAT [21,22,23,24]. Among the several types of reported metal and metal oxide-based nanozymes, cerium-based nanomaterials have been researched in detail regarding the mechanisms of their antioxidant effects. Generally, the antioxidant effects of nanoceria involve a reaction between hydrogen peroxide and Ce^4+^, in which hydrogen peroxide is degraded to molecular oxygen and Ce^4+^ is reduced to Ce^3+^. Interestingly, Ce^3+^ can be oxidized by another hydrogen peroxide and returned to the Ce^4+^ state. In this way, the cycle of Ce^4+^/Ce^3+^ is created, which is similar to the catalytic reaction of natural CATs (Figure 2) [25,26,27,28]. The cycling between Ce^4+^ and Ce^3+^ oxidation states in the presence of hydrogen peroxides can be summarized as the following reaction:

(2) $$2{\mathrm{Ce}}^{4+}+{\;H}_{2}O_{2}\to \;2{\mathrm{Ce}}^{3+}+{\;O}_{2}+2H^{+}$$

(3) $$2{\mathrm{Ce}}^{3+}+{\;H}_{2}O_{2}+2H^{+}\to \;2H_{2}O\;+2{\mathrm{Ce}}^{4+}$$

The discovery of ferromagnetic nanoparticles possessing intrinsic peroxidase-like activity was reported in 2007, and these nanoparticles are considered the first inorganic nanoparticle used as an enzyme mimetic for biomedical applications [29]. Later, ferritin–platinum nanoparticles were observed to have CAT-like activity in basic and neutral pH conditions by Nie’s group in 2011 [30]. They found that ferritin–platinum nanoparticles could facilitate the decomposition of hydrogen peroxide to generate oxygen and water, which is similar to natural CAT activity. The decomposition of hydrogen peroxide by iron-based nanozymes at basic and neutral pH levels can be summarized as follows [31]:

(4) $${\mathrm{Fe}}^{3+}+{\;H}_{2}O_{2}\to {\;\mathrm{FeOOH}}^{2+}+{\;H}_{2}O$$

(5) $${\mathrm{FeOOH}}^{2+}\to {\;\mathrm{Fe}}^{2+}+{\;\mathrm{HO}}_{2}{}^{⦁}$$

(6) $${\mathrm{Fe}}^{2+}+{\;H}_{2}O_{2}\to {\;\mathrm{Fe}}^{3+}+{\mathrm{HO}}^{-}{\;\mathrm{OH}}^{⦁}$$

(7) $${\mathrm{HO}}_{2}{}^{⦁}\to {\;H}^{+}+{\;O}_{2}^{-}$$

(8) $${\mathrm{OH}}^{⦁}+{\mathrm{HO}}_{2}{}^{⦁}/O_{2}^{-}\to {\;H}_{2}O\;+{\;O}_{2}$$

Although the CAT-like mechanisms focusing on the substrates and products in the catalytic process have been described, our knowledge of the dominating surface structures of nanozymes is still limited. Recently, Fan and co-workers reported the CAT-like activity of ferrihydrite (Fe_5_HO_8_) and demonstrated that the abundant iron-associated hydroxyl groups on the surface of Fe_5_HO_8_ have a critical influence on hydrogen peroxide decomposition [32]. The catalytic process of Fe_5_HO_8_ can be described with three main steps. First, hydrogen peroxide is adsorbed on the surface of Fe_5_HO_8_. Second, the base-like decomposition of hydrogen peroxide is then catalyzed by Fe_5_HO_8_, releasing Fe_5_HO_8_ with an H-vacancy. Third, the Fe_5_HO_8_ with an H-vacancy degrades another hydrogen peroxide through acid-like decomposition. The three steps of hydrogen peroxide decomposition by Fe_5_HO_8_ can be summarized as follows:

(9) $${\mathrm{Fe}}_{5}{\mathrm{HO}}_{8}+{\;H}_{2}O_{2}\to {\;H}_{2}O_{2}{}^{*}$$

(10) $$H_{2}O_{2}{}^{*}+\;H-\mathrm{OFe}\;\to {\;\mathrm{HO}}^{*}+{\;H}_{2}O^{*}+H-vacancy$$

(11) $$H_{2}O_{2}+{\;\mathrm{HO}}^{*}+H-vacancy\;\to {\;O}_{2}{}^{*}+{\;H}_{2}O^{*}+H-OFe$$

$$\left(*\right):species\;adsorbed\;on\;the\;surface\;of\;Fe_{5}HO_{8}$$

As a whole, the mechanism of CAT-like nanozymes can be summarized as below:

(12) $$2H_{2}O_{2}\overset{CAT-like\;nanozyme}{\to }{\;O}_{2}+2H_{2}O$$

### 2.2. Superoxide-Dismutase-Like Activity

Superoxide radicals are key elements of ROS generated during the metabolisms of living systems as by-products. Superoxide radicals are intimately involved in oxidative stress and easily converted to other ROS, making it complicated to evaluate their pathogenic pathways [33]. High levels of ROS, especially superoxide radicals, have been implicated in cardiovascular diseases (heart failure, hypertension, diabetes, hypercholesterolemia, and atherosclerosis), Parkinson’s disease, and cancers [34,35]. As a natural antioxidant enzyme, superoxide dismutase (SOD) uses metal as a cofactor and catalyzes the dismutation of superoxide radicals to hydrogen peroxide and oxygen.

(13) $$2O_{2}^{⦁-}+2H^{+}\;\overset{Superoxide\;dismutase}{\to }\;H_{2}O_{2}+{\;O}_{2}$$

SOD is known as a major cellular defense against superoxide radicals, protecting the biological body from oxidative stress. There are three isoforms of SOD that have been found in mammals (CuZnSOD, MnSOD, and ecSOD). Each is produced by distinct genes but catalyzes the same reaction. The mechanism of action of SOD relies on the cycle of the reduction and oxidation states of redox-active transition metals, such as copper and manganese, at the active site of SOD [36,37].

(14) $$Oxidized\;metal-SOD+O_{2}^{⦁-}\;\to \;Reduced\;metal-SOD+O_{2}$$

(15) $$Reduced\;metal-SOD+O_{2}^{⦁-}+2H^{+}\to \;Oxidized-SOD+H_{2}O_{2}$$

**Figure 3:**
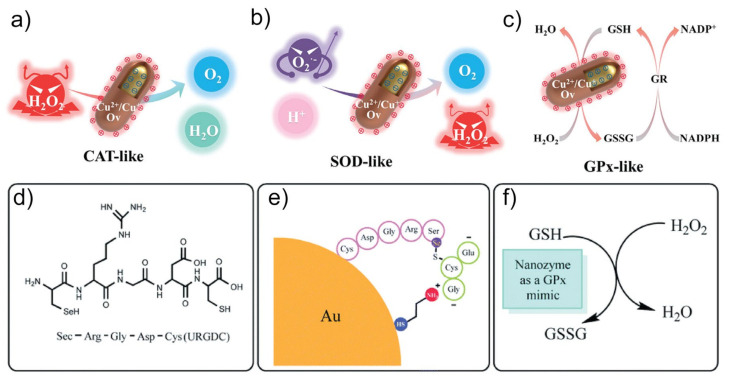
The illustration of CAT-like activity for (**a**) H_2_O_2_ decomposition, (**b**) SOD-like activity for superoxide radical scavenging, and (**c**) GPx-like activity for H_2_O_2_ decomposition. Reprinted with permission from [11]. Copyright (2023) ACS publications. (**d**–**f**) The fabrication and mechanism of selenium-containing pentapeptide (Sec-Arg-Gly-Asp-Cys)-modified gold nanozyme exhibiting GPx activity in the presence of GSH. Reprinted with permission from [56]. Copyright (2020) Royal Society of Chemistry.

Nanozymes possessing SOD-like activity play an important role in the protection against oxidative stress caused by superoxide radicals [38,39]. In 1991, a cluster consisting of 60 carbon atoms was reported as a scavenger of free radicals by Krusic et al. [40]. This research provided early proof that synthesized materials can exhibit antioxidant activity. Since then, various nanozymes have been reported to have SOD-like activity. Most superoxide dismutase nanozymes consist of transition metals (copper, iron, cerium, etc.) and elements such as nitrogen, oxygen, carbon, and sulphur. Among the various nanozymes, SOD-like cerium nanoparticles have been widely developed due to their high biocompatibility and deeply researched mechanism of action [41,42,43]. In 2007, the first cerium oxide nanoparticle exhibiting SOD-like activity was introduced by Korsvil’s group [44]. Similar to nanocerium exhibiting CAT activity, the SOD-like activity of cerium oxide is also involved in the conversion between Ce^3+^ and Ce^4+^. The change in the oxidation states of cerium oxide generates oxygen vacancies in the crystal lattice structure by giving out oxygen and electrons. The oxygen vacancies play a key role in the exhibition of SOD-like activity, allowing cerium nanoparticles to uptake or release oxygen. The presence of Ce^3+^ is a result of oxygen vacancy, and thus the high Ce^3+^/Ce^4+^ ratio provides more oxygen vacancies, which increases the SOD-like activity (Figure 3) [45,46]. The dismutation of superoxide radicals by cerium nanoparticles is displayed below:

(16) $$O_{2}^{⦁-}+{\;\mathrm{Ce}}^{4+}\;\to {\;\mathrm{Ce}}^{3+}+{\;O}_{2}$$

(17) $$O_{2}^{⦁-}+{\;\mathrm{Ce}}^{3+}+2H^{+}\to {\;\mathrm{Ce}}^{4+}+{\;H}_{2}O_{2}$$

Similar to cerium, other transition metals, such as copper (Cu) [47], gold (Au) [48], iron (Fe) [49], manganese (Mn) [50], platinum (Pt) [51], cobalt (Co) [52], and silver (Ag) [53], have been used as the main elements to fabricate nanozymes that exhibit SOD-like activity. Generally, a superoxide radical consists of a Brønsted base with p*K*_b_ = 9.12 [54], and thus, superoxide radicals can capture protons from H_2_O to form HO_2_^•^ and HO^−^. The adsorption of HO_2_^•^ on the facets of Au, Ag, Pd, and Pt can trigger the conversion of HO_2_^•^ to O_2_ and H_2_O_2_ [55].

Generally, the mechanism of SOD-like nanozymes can be summarized as below:

(18) $$2O_{2}^{⦁-}+2H^{+}\;\overset{Superoxide\;dismutase}{\to }\;H_{2}O_{2}+{\;O}_{2}$$

### 2.3. Glutathione-Peroxidase-Like Activity

Glutathione peroxidase (GPx) is involved in the termination of the ROS pathway, resulting in a decrease in oxidative stress [57,58,59]. Similar to natural CAT, GPx catalyzes the degradation of H_2_O_2_ to H_2_O and contributes to the cellular antioxidant activity of many organisms. GPx contains seleno-cysteine in its active site, in which selenol (ESeH) degrades H_2_O_2_ to H_2_O through a redox reaction and is oxidized to form selenenic acid (ESeOH). ESeOH then undergoes redox reactions with two reduced glutathiones (GSHs) to recover to ESeH. In this redox cycle, two GSHs are oxidized to form two glutathione disulfides (GSSG) [60,61,62].

Similar to natural GPx, nanozymes exhibit glutathione-peroxidase-like activity using two GSHs to generate redox cycles for H_2_O_2_ decomposition [63,64,65,66]. As a typical example, orthorhombic V_2_O_5_ nanocrystals catalyze the decomposition of H_2_O_2_ in the presence of GSH [64,67]. The glutathione-peroxidase-like activity of V_2_O_5_ involves the interaction of hydrogen atoms in H_2_O_2_ with the oxygen atoms of the V=O and V−O−V groups, whereas one of the oxygen atoms in H_2_O_2_ interacts with vanadium atoms. After reacting with the first H_2_O_2_, V=O is converted into a V-peroxide intermediate, which is then converted into V−OH by reacting with GSH. The V−OH reacts with the second H_2_O_2_ and GSH to return to the V=O form.

Other nanomaterials exhibit glutathione peroxidase-like activity, such as manganese (II, III) oxide [68], Cu_x_O nanoparticles [69], ultrasmall Cu_5.4_O nanoparticles [70], citrate functionalized Mn_3_O_4_ nanoparticles [71], and Pt/CeO_2_ nanozymes [72]. All nanozymes mimicking glutathione peroxidase use GSH as one of the substrates to perform their catalytic activity. GSH acts as a reductant and can nucleophilically attack the peroxide bond of peroxide species to form GSSG [67]. Zhang et al. synthesized a selenium-containing pentapeptide (Sec-Arg-Gly-Asp-Cys)-modified gold nanozyme exhibiting GPx activity in the presence of GSH [56]. In this system, gold nanoparticles served as a scaffold that decreased the mobility and constrained the conformation of the peptides. The synthesized nanozyme exhibited GPx activity following an ordered mechanism in which the reaction requires multiple substrates to react sequentially with the enzyme before releasing the first products. Meanwhile, the free peptides follow a ping-pong mechanism in which the first substrate binds to the enzyme and releases the first product before binding the second substrate.

## 3. Manipulation of Nanozyme Activities for Antioxidant Effects

### 3.1. Size

It is well-known that the catalytic activity of nanozymes is affected by the size of the materials. For example, cerium oxide nanoparticles with a size of 4.2 nm have been reported to have higher antioxidant activity than the same nanoparticles with a size of 14 nm [73]. Baldim et al. reported that the size of cerium oxide nanoparticles is almost inversely proportional to the SOD-like activity [13]. To explain, a decrease in the particle size results in an increase in the Ce^3+^/Ce^4+^ ratio on the surface of particles, which increases the number of defects caused by oxygen vacancy. Oxygen vacancy plays a key role in SOD-like activity, in which a higher number of oxygen vacancies provide more active sites for reactions with substrates [42,46,74,75]. As a result, cerium oxide nanoparticles with a size of ≈5 nm exhibited the highest SOD-like activity, followed by ≈8 nm, ≈23 nm, and ≈28 nm, respectively. Similar to cerium nanoparticles, carbon dot nanozymes with a size of 2 nm and a large specific surface area also provided abundant binding and active sites for catalytic reactions resulting in an increase in SOD-like activity [76]. Similarly, CeVO_4_ nanorods with sizes of ≈50 nm, 100 nm and 150 nm exhibited SOD-like activity of 4.12 ± 0.19, 3.93 ± 0.20, and 2.57 ± 0.07 ng/µL, respectively [77].

The CAT-like and GPx-like activity of nanozymes is also affected by the size of the materials. At a cerium concentration of 200 µM, the CAT-like activities of cerium nanoparticles with particle sizes of 23 and 28 nm were found to be lower than those with particle sizes of 4.5 and 7.8 nm [13]. Interestingly, Zhang et al. synthesized a single-atom nanozyme of RhN_4_ with a 20-fold higher affinity for CAT-like activity compared to natural CAT [65]. CoO nanoparticles with sizes between 25 and 30 nm exhibited CAT-like activity of 19.01 U/mL [78]. A single-atom nanozyme, VN_4_, showed a 7-fold higher GPx-like activity than natural GPx [65]. Gold nanoparticles with an average size of about 20 nm coupled with selenium-containing pentapeptide (Sec-Arg-Gly-Asp-Cys) can enhance GPx activity about 14 times compared to free selenopeptide [56]. Ultrasmall Ru nanoparticles with a size of ≈2 nm increased surface-oxidized Ru atoms, hence exhibiting a higher antioxidant activity than medium-sized (≈3.9 nm) and large-size (≈5.9 nm) Ru nanoparticles [79]. In general, the smaller the nanozyme is, the higher the level of enzyme activity it has.

### 3.2. Morphology

**Figure 4:**
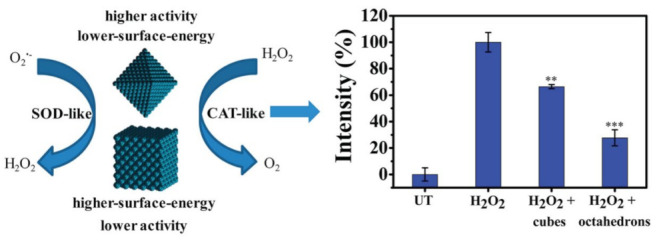
Effect of morphology on the SOD-like and CAT-like activities of Pd nanocrystals. Quantitative analysis of the ROS levels by flow cytometry. Data are represented as the mean fluorescence intensity. *p*-values compared to H_2_O_2_-treated cells were calculated by Student’s *t* test: ** *p* < 0.01, *** *p* < 0.001. Reprinted with permission from [85]. Copyright (2016) ACS publications.

Previous studies have demonstrated that the morphology of nanomaterials highly affects the catalytic performance. The morphology of nanomaterials is closely related to their specific surface area, pore size, and volume [42,80,81,82]. Most nanozymes with large specific surface areas have more exposed active sites on their surface, resulting in an increase in catalytic activity, including antioxidant activity. For example, Yang et al. synthesized cerium-based nanomaterials with different morphologies, such as nanoclusters, nanoparticles, and nanochains [83]. Among them, cerium nanoclusters showed the highest level of SOD-like activity, and cerium nanochains exhibited higher SOD-like activity than cerium nanoparticles at cerium concentrations ranging from 5 to 100 mg/L. Singh et al. compared the SOD-, CAT-, and GPx-like activity of Mn_3_O_4_ nanozymes with different shapes, such as a flower-like morphology, flake-like morphology, hexagonal plates, polyhedrons, and cubes [84]. The flower-like Mn_3_O_4_ nanozymes showed the highest SOD-, CAT-, and GPx-like activity, followed by the flake-like Mn_3_O_4_ nanozymes. The specific surface area of the flower-like Mn_3_O_4_ nanozyme was 97.7 m^2^/g, higher than that of other morphologies. Ge et al. reported that (111)-faceted Pd octahedrons with low surface energy showed higher antioxidant activity than (100)-faceted nanocubes with high surface energy (Figure 4) [85]. Mn/Zr-co-doped CeO_2_ tandem nanozymes with hollow mesopores possessed SOD-like and peroxidase-like activity, while their CAT-like activity was inhibited [86]. VO_2_ nanofibers have been reported that possess higher peroxidase-like activity than VO_2_ nanosheets and VO_2_ nanorods [87]. Co_3_O_4_ nanozymes with different morphologies exhibited different levels of CAT-like activity following the order Co_3_O_4_ nanoplates > Co_3_O_4_ nanorods > Co_3_O_4_ nanocubes [88]. In general, nanozymes with large specific surface areas, including flower-like shapes, nanoclusters, nanofibers, and mesopores, tend to exhibit higher enzyme activities.

### 3.3. Composition

**Figure 5:**
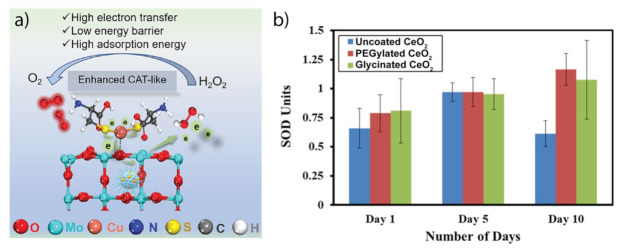
(**a**) Illustration of nanocomposite consisting of multiple elements increasing CAT-like activity. Reprinted with permission from [97]. Copyright (2023) ACS publications. (**b**) SOD-like activity of PEGylated, glycinated, and uncoated cerium nanoparticles. Reprinted with permission from [43]. Copyright (2019) ACS publications.

The enzyme-like activity of a nanozyme can be economically and efficiently regulated by adjusting the proportion and design of its scomponents [21,48,89]. One of the most effective strategies to enhance the enzymatic activity of a nanozyme is a combination between metals and organic or inorganic materials [90,91,92,93,94]. For example, polyvinylpyrrolidone–platinum–copper nanoparticle clusters (PVP-PtCuNCs) were synthesized by Liu et al. and exhibited 10-fold higher SOD-like activity and 4-fold higher CAT-like activity than PVP-PtNCs [51]. As another example, apoferritin was used as a cage to limit the growth field of and confine Au–Ag nanoparticles in a homogeneous distribution [53]. As a result, the wrapped Au–Ag nanoparticles inside apoferritins possessed 6 × 10^4^ times higher SOD-like activity than that of unwrapped Au–Ag nanoparticles. Generally, Au nanoparticles alone did not exhibit SOD-like activity, even though the concentration of Au nanoparticles was very high (300 µg/mL) [21]. However, Au core/Ce shell-based nanozymes exhibited SOD-like activity at a wide range of pH values (2−11) and temperatures up to 90 °C. Another nanocomposite consisting of Au, Cu, and cysteine (Au-Cu-Cys) showed three times higher IC_50_ values for SOD-like activity than did the natural SOD enzyme [95]. Matysik et al. conjugated natural SOD on multimetallic nanocomposites, such as ZnO–MnO, ZnO–CuO, and ZnO–MnO–CuO [96]. The SOD-like activities of such nanocomposites were ranked as ZnO–MnO+SOD > ZnO–CuO+SOD > ZnO–MnO–CuO+SOD > SOD enzyme. Zhou et al. fabricated a nanocomposite containing polyvinylpyrrolidone, a Cu single-atom nanozyme, L-cysteine, and MoO_x_ nanoparticles (denoted as MCCP) (Figure 5a) [97]. MCCP achieved impressive CAT-like activity, which was 138-fold stronger than that of typical MnO_2_.

Curcumin and graphene oxide have become the focal point of cancer studies for their strong antioxidant activity, which correlates with their anticancer property. One of the limitations of curcumin and graphene oxide is they may cause severe damage to not only cancer cells but also normal cells because of their low selectivity. In response, Al-Ani et al. used curcumin and graphene oxide along with biocompatible gold nanoparticle functionalization (CAG) to enhance their selectivity [98]. As a result, the CAG nanocomposite enhanced the selectivity index by ≥30% for SW-948 and ≥90% for HT-29 cancer cells as compared to traditional reduced graphene oxide–gold nanoparticles. The high selectivity of antioxidant nanozymes is critical for clinical applications because it can minimize toxicity.

In terms of CAT-like activities, Zhang et al., compared the enzymatic activities of ten different iron oxide nanomaterials, including 2-line ferrihydrite, 6-line ferrihydrite, akageneite, feroxyhyte, goethite, lepidocrocite, schwertmannite, maghemite, magnetite, and hematite [32]. Among the ten mentioned iron-based nanomaterials, 2-line ferrihydrite possessed the highest CAT-like activity. The CAT-like activities of other iron-based nanomaterials were ranked as 6-line ferrihydrite > feroxyhyte > other iron oxide nanomaterials. The iron-based nanomaterials possessing hydroxyl groups in their stoichiometric composition (2-line ferrihydrite, 6-line ferrihydrite, and feroxyhyte) tended to exhibit higher CAT-like activity than iron-based nanomaterials possessing no hydroxyl groups (hematite, maghemite, and magnetite). It was reported that hydroxyl groups provided the sites for ion exchange to occur on the surface of metal oxides, and thus, the presence of hydroxyl groups could greatly enhance CAT-like activity [99,100]. However, goethite with a low specific surface area still exhibited low CAT-like activity despite its high surface hydroxyl site density. Akageneite possessing a low surface hydroxyl site density and a high specific surface area also exhibited low CAT-like activity.

Most nanoparticles are vulnerable to aggregation, leading to a decrease in their antioxidant activity. Zhou et al., addressed the problem of aggregation using 2D graphdiynes (GDY) to immobilize ultrasmall CeO_2_ nanoparticles as well as enhance CAT-like activity [101]. As a result, GDY-CeO_2_ nanocomposites showed a 4.2-fold greater rate constant in H_2_O_2_ decomposition than CeO_2_ nanoparticles. Remarkably, a nanozyme consisting of a V_2_O_5_ nanowire, MnO_2_ nanoparticles, and dopamine was designed and synthesized to mimic multiple oxidant enzymes, such as SOD, CAT, and GPx [102]. In this nanocomposite model, V_2_O_5_ nanowires possessed GPx-like activity, while MnO_2_ nanoparticles possessed SOD- and CAT-like activities. Dopamine was used to assemble the V_2_O_5_ and MnO_2_ nanomaterials. The combination between V_2_O_5_, MnO_2_, and dopamine exhibited significantly higher ROS scavenging capacity than V_2_O_4_ and MnO_2_ nanomaterials. As another example of nanocomposite exhibiting multiple antioxidant enzymes, copper–tannic acid nanozymes were reported to be a thermostable and highly active SOD mimic and CAT mimic [103]. The copper–tannic acid nanozymes with a concentration of 1 µg/mL exhibited almost the same SOD-like activity compared to the natural SOD enzyme with a concentration of 10 U/mL. However, the copper–tannic acid nanozyme could degrade superoxide radicals at a wide range of temperatures (25–80 °C), while natural SOD could not exhibit enzymatic activity at temperatures higher than 40 °C. The CAT-like activity of copper–tannic acid was strongly stable even at 80 °C.

As a whole, the composition of antioxidant nanozymes can highly influence antioxidant performance. Adjusting their proportions and regulating their chemical components, such as breaking the working pH and temperature limits, enabling multi-target function, avoiding self-aggregation, etc., improves the current limitations of antioxidant nanozymes and controls their enzyme-like properties.

### 3.4. Surface Modification

Surface modification is another factor that highly affects antioxidant activity because most antioxidant reactions occur on the surface of nanomaterials. Numerous studies have reported that surface properties such as thickness, surface charges, and functional groups are important factors in manipulating the activity of nanozymes [104,105,106]. For example, gold nanoparticles capped with *N*-acetylcysteine (Au-NAC) achieved an ultrasmall size with an average diameter of 2 nm and possessed much higher SOD-like activity than free *N*-acetylcysteine and gold nanoparticles [107]. A bacteria-like nanozyme was fabricated by decorating ultrasmall CeO_2_ nanoparticles into a dendritic mesoporous silica-coated Bi_2_S_3_ (Ce-Bi@DMSN). Then, the Ce-Bi@DMSN was coated with polyethylene glycol (PEG) [108]. CeO_2_ endowed the nanozyme with high CAT-like activity. Bi@DMSN acted as a stabilizer to prevent CeO_2_ from precipitation. The PEG-coated surface endowed the nanocomposite with high hydrophilicity. In addition, it was reported that glycinated and PEGylated ceria nanoparticles exhibited 1.75–1.9 times higher SOD-like activity than uncoated ceria nanoparticles (Figure 5b) [43]. The high physiological stability, biocompatibility, and monodisperisty of nanozymes can be achieved through coating with polyvinylpyrrolidone (PVP) [109], amine-terminated PAMAM dendrimer [110], mercaptoundecanoic acid (11-MUA) [111], PEG [112,113], etc.

Strain effects are a neoteric approach for enhancing the antioxidant activity of nanozymes. Strain effects arise from the torsional angles, distortion of chemical bonds, or mismatched lattices [114,115,116]. Han et al. generated a strained Mn_3_O_4_ layer on CeO_2_ nanoparticles [117]. O 1s XPS spectra results indicated that the ratio of oxygen defects to lattice oxygen in the strained Mn_3_O_4_ on CeO_2_ nanozymes (0.85) was higher than that of CeO_2_ nanozymes (0.60), leading to the enhancement of antioxidant activities involved in SOD- and CAT-like activity.

### 3.5. Modification with Metal–Organic Framework

Recently, metal–organic framework (MOF) nanozymes emerged as a huge, remarkable class of functional materials, especially for mimicking antioxidant activity [118,119,120,121]. MOFs are porous materials that are obtained through the self-assembly of organic ligands and metal nodes. One of the most attractive features of MOF nanozymes is their great synthetic tunability, which allows fine chemical and structural control. Depending on their specific purposes, different MOF nanozymes can be rationally designed with properties such as stability, porosity, and particle morphology [122]. It has been reported that a high Ce^3+^/Ce^4+^ ratio increases SOD-like activity [45,46]. However, two monovalent Ce-based MOF constructed by 1,3,5-benzenetricarboxylic acid (Ce^III^BTC and Ce^IV^BTC) showed unexpected SOD-like activity in which Ce^IV^BTC exhibited higher SOD-like activity than Ce^III^BTC. Furthermore, both Ce^III^BTC and Ce^IV^BTC showed high selectivity toward superoxide radical elimination [121]. Magnesium gallate MOF with micropores exhibited remarkable antioxidant activity as well as biocompatibility [123]. Copper-based MOF was synthesized to decompose H_2_O_2_ and considered a CAT mimic with high catalytic activity [124]. For mimicking glutathione peroxidase, selenium-containing molecules (PhSeBr) were grafted to a Zr(IV)-based UiO-66-NH_2_ framework. In this mimic of the glutathione peroxidase system, PhSeBr acted as a donator, while a Zr(IV)-based UiO-66-NH_2_ framework with a high surface area and uniform porosity provided more catalytic active centers, resulting in a high enzyme-like activity [125]. Sang et al. synthesized PZIF67-AT nanoparticles through coordination between a 2-methylimidazole organic linker and cobalt ions [126]. The SOD-mimicking activity of the synthesized PZIF67-AT nanoparticles endowed them with the ability to efficiently produce H_2_O_2_ from superoxide radicals. Simultaneously, PZIF67-AT suppresses CAT and GPx activity, preventing the transformation of H_2_O_2_ to water. In this way, PZIF67-AT increases H_2_O_2_ accumulation, which facilitates Fenton reaction-based chemodynamic therapy. Wang et al. prepared a self-assembled photodynamic therapy nanoagent (OxgeMCC-r single-atom enzyme) consisting of single-atom ruthenium doped into Mn_3_[Co(CN)_6_]_2_ MOF and encapsulated with chlorin e6 [127]. The ruthenium serves as an active center for the conversion of H_2_O_2_ into O_2_, endowing oxgeMCC-r nanozyme with a high level of CAT-like activity. The oxgeMCC-r nanozyme could selectively accumulate in the tumor sites to increase the performance of photodynamic therapy. A cerium-based metal–organic framework (Ce-MOF) was synthesized employing cerium as the active center and 4,4′,4′′-nitrilotribenzoic acid as the linker [128]. With CAT-, SOD-, and peroxidase-like enzymatic activities, Ce-MOF could effectively eliminate fungi such as *Rhodotorula glutinis*, *Aspergillus terreus*, *Aspergillus flavus*, *Aspergillus niger*, and *Candida albicans*. In brief, the porous structure and multiple channels of MOFs assist the contact of ROS with the active catalytic sites of MOF nanozymes. The antioxidant activity of MOF nanozymes can be controlled by changing the size of pores, and different enzyme-like activities can be rationally designed by changing the combination of organic ligands and metal nodes. MOF-based antioxidants and their derivatives hold great potential for replacing natural enzymes thanks to their notable features, including their highly specific surface area, the ability to adjust their pore size, and their tunable porosity, as shown in Table 1. Moreover, MOF-based antioxidants demonstrate high thermal stability, flexibility, biocompatibility, and versatile functionality, making them become a feasible strategy for scavenging ROS.

## 4. Applications

### 4.1. Applications in Medicine and Healthcare

Antioxidant nanozymes provide a wide variety of benefits to the medical field, and they have been proven for their therapeutic potential, especially for oxidative damage-related pathological diseases. The excess ROS level in our body can be a deleterious effect that leads to various types of cancer by inducing genetic mutations, raising abnormal protein functions, and promoting tumor growth [129,130]. In response, various antioxidant nanozymes have been investigated to balance ROS levels for cancer prevention. Ismail et al. analyzed the potential of platinum nanoparticles to decrease oxidative stress conditions in lung cancer. The data supported that epithelial lung cancer cells did not survive when they were exposed to platinum nanoparticles for 3 h [131]. Cerium oxide nanoparticles also possess anticancer properties by inducing antioxidant activity. Cerium oxide nanozymes with mimetic antioxidant activities, such as CAT-like and SOD-like activities, demonstrate ROS regulation abilities, which can be used to enhance antitumor therapies, including photodynamic and photothermal therapy [28]. It is worth noting that cerium oxide nanoparticles protect cells against oxidative stress at the neutral pH of healthy cells, though they can also induce oxidative stress in the acidic conditions of cancer cells [132,133]. Due to their high flexibility, cerium oxide nanozymes can be used as cytotoxic drugs to kill cancer cells and as protective agents for normal cells.

Age-related diseases, including Alzheimer’s and Parkinson’s disease, are closely associated with ROS accumulation [134,135,136]. According to a recent study, MoS_2_ nanosheets have been investigated regarding their SOD-like and CAT-like activity to quench ^•^NO, O_2_^•–^ and ^•^DPPH free radials [137]. Ceria (CeO_2_) additionally play an SOD-like role by shuttling between the Ce^3+^ and Ce^4+^ states of the mitochondria and certain pathways in Alzheimer’s disease mouse models [34,138,139]. With their ROS-scavenging activity, ceria also take part in therapeutic applications for Parkinson’s disease and depression [140,141].

Moreover, the supporting role of antioxidant-like nanozymes has been demonstrated in research on the circulatory system, inflammation, and metabolic diseases. A study on hydrogel/nanoparticles-based stem cells proved the ROS-suppressing ability of fullerenol by activating the ERK and p38 pathways [142]. Another report suggested the anti-inflammation action of graphene oxide via controlling ROS generation during the M1 macrophage polarization process in the cardiac infraction region [143]. In addition, certain nanozymes, such as copper-based nanoparticles, ceria, and Mn_3_O_4_ [70,144,145], have been applied for the therapeutic examination of injuries. Since oxidative stress leads to many metabolic disorders, the ROS-scavenging effect of nanoparticles has been applied to the treatment of various diseases as well. To be specific, polydopamine nanoparticles, fenozyme, and self-cascade MoS_2_ nanozymes were investigated to improve periodontal disease, cerebral malaria, and hepatic fibrosis, respectively [146,147,148].

**Figure 6:**
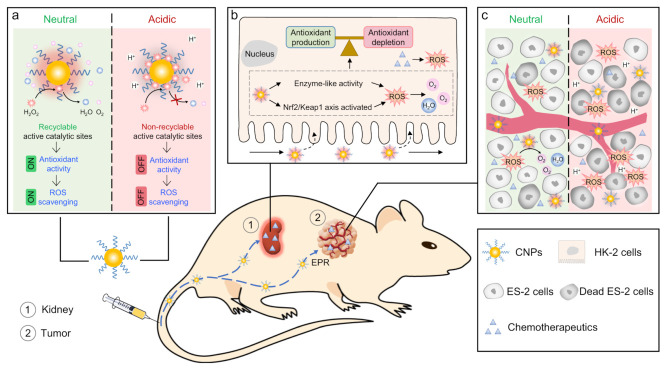
Schematic illustration of the design and characterization of catalytic-activity-tunable ceria nanoparticles (CNPs) that protect against chemotherapy-induced acute kidney injury (AKI). (**a**) CNPs switch their antioxidant activity in a pH-dependent manner. (**b**) CNPs scavenge the excessive chemotherapeutics-induced ROS. (**c**) The acidic tumor microenvironment suppresses their ROS scavenging capability without causing interference with the chemotherapeutic efficiency. Reprinted with permission from [156]. Copyright (2021) Springer Nature.

Hepatic ischemia-reperfusion injury (IRI) is a pathophysiological process that disturbs liver metabolism and damages other tissues and organs. IRI mainly results from an excess of ROS promoting multiple deleterious processes in DNA, proteins, and lipids, which cause cell death, inflammation, and liver dysfunction and failure. Due to their ROS scavenging ability, hydrophilic carbohydrate-derived nanoparticles (C-NPs) were synthesized and used as nanoantioxidants to prevent hepatic IRI [149]. C-NPs possessed excellent properties, such as nontoxicity, good colloidal stability, ROS scavenging ability, selective delivery to the liver, good circulation lifetime, and slow degradability, all of which are required to maintain a healthy liver status. Liu et al. investigated melanin nanoparticles for antioxidative therapy to protect the brain from ischemic stroke [150]. Melanin nanoparticles exhibiting broad antioxidant activities against O_2_^•−^, ^•^OH, H_2_O_2_, ^•^NO, and ONOO^−^ can significantly decrease the severity of the brain injury caused by an ischemic stroke. Other studies used antioxidant nanozymes to alleviate acute kidney injury (AKI), another oxidative stress-related pathological disease [107,151,152]. The adopted adjuvant treatment of AKI uses small antioxidants such as amifostine, ι-carnitine, and *N*-acetylcysteine [153,154,155]. However, these antioxidants have a low ability to target the kidney and large sizes, which decreases their effectiveness for AKI treatment and makes them a challenge to accumulate in renal tissue. Antioxidant nanozymes with diverse surface modifications, sizes, and shapes allow them to specifically target the kidney and easily accumulate in renal tissue. Cerium oxide nanozymes exhibiting multiple antioxidant activities, including SOD- and CAT-like activities, provided efficient protection of renal tissue against oxidative stress caused by an excess of H_2_O_2_ in vitro and in vivo (Figure 6) [151,156]. Other metal and metal oxide nanozymes, such as platinum [157], gold [107], iridium [158], copper [70], and manganese [159] nanozymes, showed significant improvements in AKI treatment.

### 4.2. Applications in Diagnostics and Analytics

In addition to the application of antioxidant nanozymes in medicine, antioxidant nanozymes also provide broad benefits for diagnostic and analytic purposes. With the rapid development of biosensors, antioxidant nanozyzmes have been found to be promising tools in targeted substance detection. Combinations between nanozymes and conventional detection techniques, such as electrochemical-, colorimetric-, and fluorescence-based analysis, offer opportunities to develop new strategies for diagnostics and analytics [160,161,162]. The past decade has witnessed the broad application of nanozyme-based biosensors to detect specific targets. For example, Liang et al. introduced polyethyleneimine-coated nanocubes (PEI NCs) to detect illegal additives [163]. This system provided a pH-switchable dual mode for detecting rosiglitazone, which can exhibit catalase-like and peroxidase-like activity in alkaline and acidic condition, respectively. In alkaline conditions, the catalase-like activity of PEI NCs reduced the fluorescence intensity of 4-chloro-1-naphthol in the presence of rosiglitazone. In acidic conditions, the peroxidase-like activity of PEI NCs triggered a colorimetric reaction that changed the color of 3,3′,5,5′-tetramethylbenzidine (TMB) in the presence of rosiglitazone. The CAT-like and SOD-like activity of antioxidant nanozymes, such as Co_3_O_4_ [164], vanadium-based nanozymes [165], and copper nanozymes [166], endows them the ability to detect biomarkers, microbiological pathogens, and toxic compounds in the presence of H_2_O_2_ by changing the color of TMB. In general, antioxidant nanozymes catalyze the oxidation of TMB, which changes TMB’s color from colorless to blue in the presence of H_2_O_2_. However, the presence of targets interrupts the oxidation of TMB, preventing this color change. This strategy was used to detect several targets, such as glutathione [164], superoxide anion [165], ascorbic acid [166], and amyloid-β peptide [167].

Antioxidant nanozymes mainly contribute to electrochemical analysis by exhibiting SOD-like activity to analyze superoxide radicals, which are highly related to the multi-stage cancerization of healthy tissue, diabetes, Parkinson’s disease, etc. [168,169]. In this strategy, SOD-mimicking nanozymes, such as CeO_2_ [170], Mn_x_(PO_4_)_y_ [171], CeO_2_–TiO_2_ [172], and Co_3_(PO_4_)_2_ [173], scavenge superoxide radicals to produce O_2_ on the electrode of an electrochemical sensor. The O_2_ accumulation on the electrode increases the current density to a higher level. By changing the electric current obtained at the electrode in the presence of the target, the electrochemical sensor not only detects superoxide radicals but also quantifies them. The integration of SOD-like nanozymes and electrochemical sensors provides fast, precise, sensitive, and selective analytical tools for superoxide radical analysis.

## 5. Conclusions and Future Perspectives

In this review, we summarized recent nanozymes with antioxidant effects, focusing on the mechanisms and neoteric approaches for activity regulation. Generally, nanozymes exhibit antioxidant activity by mimicking three main natural enzymes: catalase, superoxide dismutase, and glutathione peroxidase. Catalase- and glutathione peroxidase-mimicking nanozymes decompose H_2_O_2_, while superoxide dismutase-mimicking nanozymes catalyze thesuperoxide radical decomposition. In addition, the enzymatic activities of nanozymes can be regulated through the modification of their size, morphology, surface, and composition, as well as modification with MOF. The modification of size and morphology mainly manipulates antioxidant activity because size and morphology strongly affect the specific surface area. Similarly, the stability, biocompatibility, selectivity, and multi-target ability of antioxidant nanozymes can be manipulated through the modification of their surface and composition, as well as through MOF. Although antioxidant nanozymes offer many advantages in the improvement of natural antioxidant enzymes, there are still limitations that need to be further addressed. First, numerous nanozymes are poorly biocompatible as compared to natural enzymes because of the use of toxic agents in nanozyme fabrications. Therefore, the green synthesis of antioxidant nanozymes should be investigated to extend the application of antioxidant nanozymes, especially in clinical fields. Second, antioxidant nanozymes can become pro-oxidants, which have the opposite action of antioxidants, causing oxidative stress depending on the internal environment. For example, a nanozyme can act as an antioxidant at a high pH level but can act as a pro-oxidant at a low pH level. Fortunately, this property can be used to specifically target oxidative-stressed sites, which are mainly acidic. On the other hand, antioxidant nanozymes can produce ROS when they meet acidic but healthy body parts, such as the stomach, skin, and large intestine. Therefore, studies on the response of antioxidant nanozymes to different areas of the human body should be thoroughly investigated, thus extending the applications of antioxidant nanozymes.

## Figures and Tables

**Table 1:** Manipulation of antioxidant nanozymes.

Factor	Nanozyme	Catalytic Activity	Outcome
Size	CeO_2_ nanoparticle (~5–28 nm) [13]	CAT, SOD	Catalytic activities are enhanced for smaller particles and for the particles with larger Ce^3+^ fractions
	Carbon dot (2 nm) [76]	SOD	SOD activity of over 10,000 U/mg
	CeVO_4_ nanorod (50–150 nm) [77]	SOD	The inhibition of formazan production by rods with sizes of 50, 100, and 150 were 4.12 ± 0.19, 3.93 ± 0.20, and 2.57 ± 0.07 ng/µL, respectively
Morphology	Ceria nanomaterial (nanocluster, nanoparticle, nanochain) [83]	SOD	SOD activities: nanoclusters > nanochains > nanoparticles
	Mn_3_O_4_ (flower-like morphology, flake-like morphology, hexagonal plates, polyhedrons, cubes) [84]	CAT, SOD, GPx	Flower-like Mn_3_O_4_ with a specific surface area of 97.7 m^2^/g exhibited the highest CAT, SOD, and GPx activities
	Co_3_O_4_ (nanoplates, nanorods, nanocubes) [88]	CAT	CAT activity: Co_3_O_4_ nanoplates > Co_3_O_4_ nanorods > Co_3_O_4_ nanocubes
Composition	PVP–PtCuNCs [51]	CAT, SOD	PVP–PtCuNCs exhibited 10-fold higher SOD-like activity and 4-fold higher CAT-like activity than PVP–PtNCs
	Au core/Ce shell-based nanozyme [21]	SOD	Can work under extreme conditions: pH (2–11) and temperatures up to 90 °C
	CeO_2_ nanoparticles immobilized on 2D graphdiynes [101]	CAT	4.2-fold greater rate constant in H_2_O_2_ decomposition than CeO_2_ nanoparticles Prevents CeO_2_ aggregation
Surface modification	AuNP capped with *N*-acetylcysteine [107]	SOD	Higher SOD-like activity than free *N*-acetylcysteine and gold nanoparticles
	PEG-coated Ce-Bi@DMSN [108]	CAT	PEG-coated surface endows the nanocomposite with high hydrophilicity
MOF	Grafting PhSeBr to a Zr(IV)-based UiO-66-NH_2_ framework [125]	GPx	PhSeBr acted as a donator, while the Zr(IV)-based UiO-66-NH_2_ framework with high surface area and uniform porosity provided more catalytic active centers, resulting in a high enzyme-like activity
	PZIF67-AT [126]	SOD	Simultaneously increased SOD activity and suppressed CAT and GPx activity. Effectively accumulated H_2_O_2_ for Fenton reaction-based chemodynamic therapy

## Data Availability

No new data were created or analyzed in this study. Data sharing is not applicable to this article.
